# A review on immunomodulatory effects of BPA analogues

**DOI:** 10.1007/s00204-023-03519-y

**Published:** 2023-05-19

**Authors:** Anja Kodila, Nina Franko, Marija Sollner Dolenc

**Affiliations:** Faculty of Pharmacy, University of Ljubljana, Aškerčeva cesta 7, 1000 Ljubljana, Slovenia

**Keywords:** BPA analogues, Immunomodulation, Immunotoxicology, Endocrine disruptors, Bisphenols

## Abstract

Bisphenol A (BPA) is a known endocrine disruptor found in many consumer products that humans come into contact with on a daily basis. Due to increasing concerns about the safety of BPA and the introduction of new legislation restricting its use, industry has responded by adopting new, less studied BPA analogues that have similar polymer-forming properties. Some BPA analogues have already been shown to exhibit effects similar to BPA, for example, contributing to endocrine disruption through agonistic or antagonistic behaviour at various nuclear receptors such as estrogen (ER), androgen (AR), glucocorticoid (GR), aryl hydrocarbon (AhR), and pregnane X receptor (PXR). Since the European Food Safety Authority (EFSA) issued a draft re-evaluation of BPA and drastically reduced the temporary tolerable daily intake (t-TDI) of BPA from 4 mg/kg body weight/day to 0.2 ng/kg body weight/day due to increasing concern about the toxic properties of BPA, including its potential to disrupt immune system processes, we conducted a comprehensive review of the immunomodulatory activity of environmentally abundant BPA analogues. The results of the review suggest that BPA analogues may affect both the innate and acquired immune systems and can contribute to various immune-mediated conditions such as hypersensitivity reactions, allergies, and disruption of the human microbiome.

## Introduction

Bisphenol A (BPA) is one of the most abundant chemicals in the plasticizer industry due to its favourable polymer-forming properties. It is most commonly used as a precursor to epoxy resins or as a building block for polycarbonate plastics and is found in numerous consumer products such as electronics, dental fissure sealants, children’s toys, personal care products, thermal paper, and in various food containers (Lin et al. 2017). In addition to BPA exposure through water, air, and dust, humans are believed to come into contact with the aforementioned contaminant primarily through food (Sciences 2015). BPA can be released from coatings and materials into food and beverages through hydrolysis or diffusion of BPA residues that remain on the polycarbonate. Food temperature, pH, processing method, and duration of exposure are also important factors affecting the amount of BPA released into the environment (Hoekstra and Simoneau 2013).

Nevertheless, studies have shown that dermal exposure to BPA is more severe than oral exposure because BPA bypasses hepatic first-pass metabolism in this way and therefore prevents the formation of the nontoxic BPA conjugate, as is the case with oral intake (Liu and Martin 2017). Dermal exposure also results in a prolonged presence of BPA in biofluids and higher concentrations of unconjugated BPA in serum (Liu and Martin 2017).

The Classification, Labelling, and Packaging (CLP) Regulation ((EC) No. 1272/2008) classifies BPA as potentially toxic to reproduction, possibly irritating to the skin and respiratory tract, and potentially harmful to the eyes (European Chemicals Agency 2022). In addition, BPA is a known endocrine disruptor whose effects have been linked to the development of several diseases, including impaired fertility, breast cancer, diabetes, obesity, cognitive impairment, and cardiovascular disease (Catenza et al. 2021; Krause et al. 2022). Due to increasing concerns about the safety of BPA, the European Commission has banned the use of BPA in the manufacture of polycarbonate bottles for infant feeding, and a limit for BPA content in thermal paper was introduced in 2016 at the suggestion of France (European Chemicals Agency 2022). In addition, EFSA drastically reduced the tolerable daily intake (TDI) for BPA from 4 µg/kg body weight (bw)/day to 0.2 ng/kg bw/day, as BPA has been shown to have adverse effects on the immune system, in particular an increase in the number of T-helper cells (Enzymes et al. 2016; Implementation of the evidence-based risk assessment for the re-evaluation of Bisphenol A: preparatory work on cross-sectional studies 2021; EFSA 2023). Industry has responded to the new laws and bans by using a variety of less-studied BPA analogues whose toxicological properties are not yet fully understood (Ye et al. 2015; Chen et al. 2016).

As with BPA, humans are most exposed to BPA analogues through dietary sources (Geens et al. 2012). In the United States (U.S.), bisphenol F (BPF) was found to be the second most prevalent bisphenol next to BPA in various foodstuffs, with a mean concentration of 0.93 ng/g wet weight (ww). In comparison, the mean concentration of BPA in the U.S. is reported to be 3 ng/g ww, indicating its dominance among bisphenols (Liao and Kannan 2013). The level of bisphenol exposure is also highly dependent on geographic location or proximity to an industrial site (Chen et al. 2016). For example, the mean estimated daily intake (EDI) of the sum of all bisphenols for adults in the U.S. is reported to be 54.6 ng/kg bw/day, while in China the levels reach 646 and 664 ng/kg bw/day for adult men and women, respectively (Liao and Kannan 2014a). Several European studies also report the presence of BPA and its analogues BPF, bisphenol S (BPS), and bisphenol B (BPB) in canned foods, which contain the highest levels of individual and total bisphenols (Liao and Kannan 2013; Grumetto et al. 2008; Alabi et al. 2014; Viñas et al. 2010). Interestingly, white mustard seeds appear to be an important dietary source of BPF, with concentrations as high as 8 mg/kg. The literature suggests that consumption of 20 g of mustard can introduce 100–200 µg of BPF into the body (Zoller et al. 2015). Bisphenols have also been detected in environmental compartments such as seawater, sediments, sewage sludge, and wastewater, with BPA remaining the most abundant, followed by BPF and BPS (Chen et al. 2016). As for human biomonitoring, studies have mainly examined urine samples, in which bisphenols are usually present in the form of conjugates. Urinary biomonitoring data provide an estimate of daily intake rates that range from 0.001 to 0.863 µg/kg/day for BPA analogues in U.S. and Asia, about an order of magnitude lower than those for BPA. At this point, it should be noted that the number of studies addressing biomonitoring of BPA analogues is much smaller than the number of similar studies with BPA, which seems to undermine the actual human exposure to the aforementioned compounds (Chen et al. 2016).

Some studies have raised concerns about the actual safety of BPA analogues due to similar structural elements such as the phenyl moiety and hydrophobic nature, which could contribute to endocrine activity (Usman and Ahmad 2019). The most common and abundant BPA analogues in the environment are listed in Table 1, where similarities and differences are clearly illustrated in comparison to the structure of BPA. Furthermore, literature states estrogen like activities of BPA analogues such as bisphenol AF (BPAF), bisphenol E (BPE), BPS, bisphenol AP (BPAP), bisphenol P (BPP), bisphenol C (BPC), bisphenol Z (BPZ), tetramethyl bisphenol A (TMBPA), and 4,4-bisphenol F (4,4-BPF), as they demonstrate estrogen receptor (ER) at concentrations of 0.001 µM to 1 µM, with BPAF exerting the strongest effect in both an agonistic and antagonistic manner (Program and Protocol for Systematic Review of Bisphenol 2015; Program and Biological activity of Bisphenol A (BPA) structural analogues and functional alternatives 2017). Rosenmai et al. (2014) showed that BPA and its analogues, more specifically bisphenol B (BPB), BPE, bisphenol F (BPF), BPS, and 4-cumylphenol (HPP), also exerted antiandrogenic effects, with EC50 values ranging from 1.9 µM (BPE) to 5.1 µM (HPP). The authors additionally suggest that the effect of bisphenol analogues on PXR (Sui et al. 2012; Sui 2014) and AhR (Ziv-Gal et al. 2013) receptors may contribute to general endocrine disruption, as activation of these receptors affects the expression of certain enzymes involved in the metabolism of endogenous hormones (Rosenmai et al. 2014). In conjunction with endocrine disruption, studies also indicate the neurotoxic, genotoxic, and cytotoxic potential of BPA analogues, as well as toxic effects on the reproductive system (Chen et al. 2016; McDonough et al. 2021a).

**Table 1:** BPA and its most common analogues included in this review article that exhibit immunomodulatory activity

Compound	Chemical structure	Application
BPA (2,2-Bis(4-hydroxyphenyl)propane)		Polycarbonate plastic, epoxy resins, children toys, medical equipment, dental sealants, electronics, food storage containers, personal hygiene products, eyeglass lenses (Lin et al. 2017; Nowak et al. 2019)
BPS (4,4'-Sulfonyldiphenol)		Thermal paper and other products involved in paper production (brochures, envelopes, food boxes and paperboards, tickets, etc.), cleaning products, phenolic resins and as an electroplating solvent (Rochester et al. 2012; Rochester and Bolden 2015)
BPAF (4,4'-(Hexafluoroisopropylidene)diphenol)		Fluoropolymers, fluoroelastomers, optical and electronic products (Liao and Kannan 2014b)
BPF (4,4'-Methylenediphenol)		Epoxy resins and coatings found in pipe linings, electrical varnishes, adhesives and industrial floors (Rochester and Bolden 2015; Liao and Kannan 2014b)

The bisphenol family is characterised by two hydroxyphenyl functionalities that can be linked by various bridges. BPA contains two methyl groups replaced by sulphur and oxygen atoms in the case of BPS and by two trifluoromethyl groups in the case of BPAF. The phenol rings in BPF are linked by a methylene linking group. The table also lists the most common applications of the above compounds

As noted above, the available literature also points to the effects of BPA exposure during development on impaired immune function, potentially increasing the risk of developing immune-related diseases such as type 1 and 2 diabetes mellitus, multiple sclerosis, systemic lupus erythematosus (SLE), breast cancer, and allergies (McDonough et al. 2021a; Xu et al. 2016; Alhomaidan et al. 2019). BPA may be able to exert its immunomodulatory effects by acting on various nuclear and nonnuclear receptors (Rogers et al. 2013), similar to the immunosuppressive effects of endogenous and exogenous estrogens, which can modulate the release of proinflammatory cytokines and regulate Th1/Th2 responses (Salem 2004). BPA can further affect the immune system through epigenetic changes by disrupting cell signalling pathways and affecting the gut microbiota (Xu et al. 2016). As the frequency of occurrence of bisphenol analogues in the environment and in human biological samples is increasing (Chen et al. 2016), this review aims to summarise data from in silico, in vitro, and in vivo studies on the immunomodulatory effects of bisphenol analogues.

## Effects of bisphenols on immune system components

### Innate immune system

Given that the acquired component of the immune system is found primarily in vertebrates, the vast majority of other organisms rely mainly on innate immunity to maintain homeostasis (Turvey and Broide 2010). Considering that large amounts of endocrine disruptors are leached into environmental waters, these substances may have a significant impact on the innate immune system of aquatic organisms (Xu et al. 2013). So far, several studies have already addressed this issue, indicating immunomodulatory effects of bisphenols on the immune system of fish (Qiu et al. 2018a, b; Qiu et al. 2016; Qiu et al. 2018c; Ji et al. 2013; Wu et al. 2011). In addition to the main function of defense against various foreign pathogens, innate immunity also plays a role in the development of various immune-mediated diseases, such as asthma, systemic lupus erythematosus (SLE), type 1 diabetes, and inflammatory bowel disease (Turvey and Broide 2010). Data on this topic are organized according to the characteristics of the cells of the innate immune system, into tissue-bound cells (such as macrophages and dendritic cells) and “motile” cells (such as monocytes, neutrophils, and eosinophils) (Moser and Leo 2010).

#### Macrophages

Macrophages are an essential component of the innate, nonspecific immune system, their main function being the phagocytosis of foreign substances. Their presence in the vast majority of tissues illustrates their diversity and, in particular, highlights their important role in the first line of defense against pathogens (Locati et al. 2019).

**Fig. 1:**
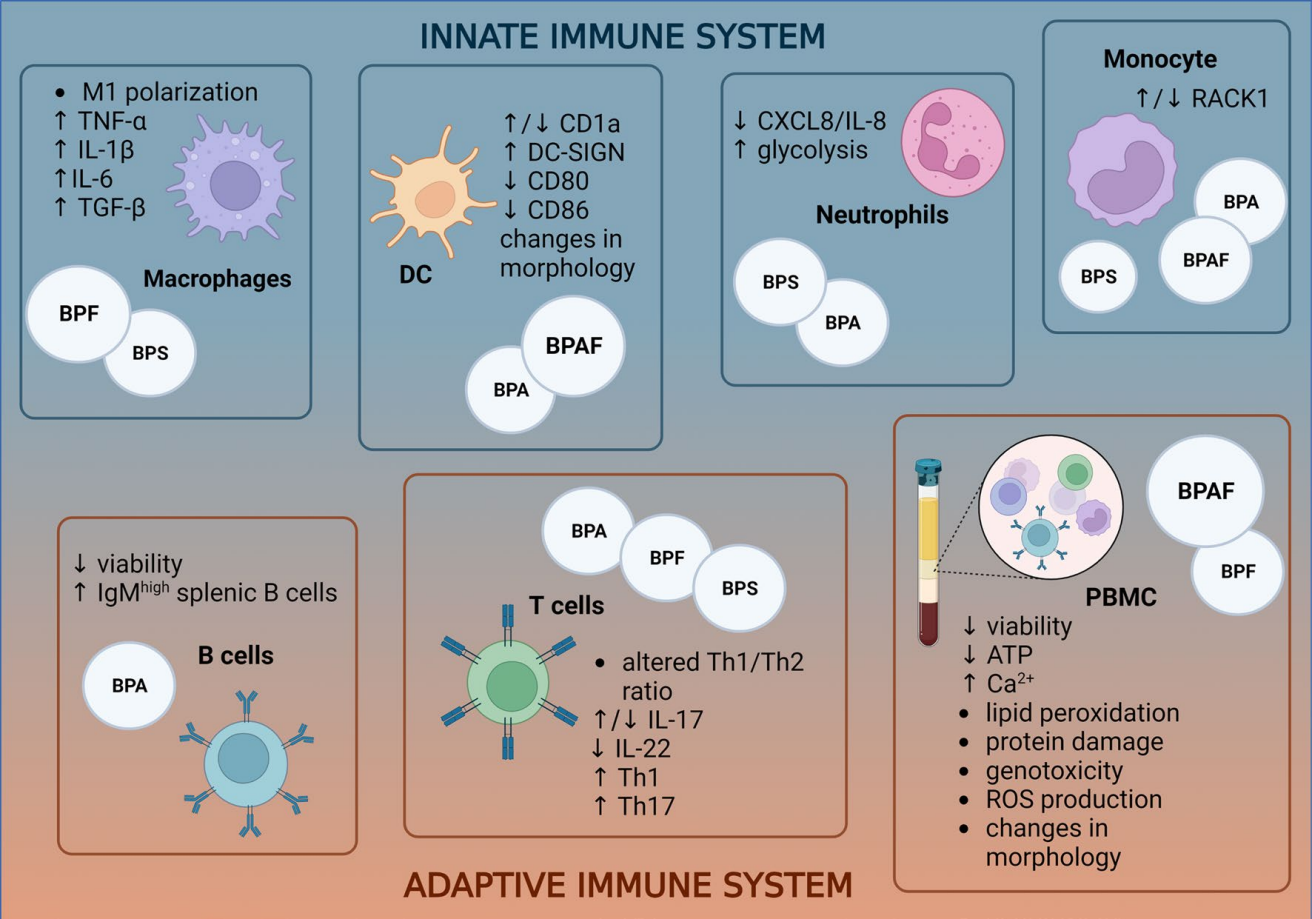
Effects of BPA and its most common analogues on various components of the immune system. The figure shows that BPA analogues affect both the innate and acquired parts of the immune system. The upward pointing arrows represent an increase in a particular parameter, and the downward pointing arrows represent a decrease (Zhao et al. 2017; Zhang et al. 2021; Švajger et al. 2016; Guo et al. 2010; Camarca 2016; Peillex et al. 2021; Buoso 2021; Malaisé et al. 2020a; Li et al. 2018a; Michałowicz et al. 2015; Mokra and Michałowicz 2015; Mokra et al. 2018; Buoso et al. 2020; Chen et al. 2018). Created with BioRender.com

The BPA analog BPF was found to promote M1 polarization of macrophages and cause a shift to a pro-inflammatory state with statistically significant increases in secretion of the pro-inflammatory cytokines TNF-α, IL -1β, and IL -6 at concentrations of 10 and 20 µM in the murine macrophage cell line RAW 264.7, as shown in Fig. 1 (Shi et al. 2020). The results are consistent with previous studies investigating another BPA analog BPS that also leads to a shift toward M1 polarization in mouse macrophages, measured as an increase in iNOS mRNA levels (Zhao et al. 2017). Similarly, BPS was also found to increase levels of cytokines such as TNF-α, IL -1β, IL -6, and TGF-β in the mouse macrophage cell line J774A.1 (Fig. 1) (Zhao et al. 2017). Zhao et al. (2017) also found that BPS -induced M1 polarization and resulting pro-inflammation are associated with alterations in the macrophage metabolome and lipidome, particularly alterations in the glycolytic pathway, as well as glutathione signalling pathways and aberrations in lipid signalling pathways (Zhao et al. 2017). Similar to BPS, the proinflammatory properties of BPF have been shown to be associated with alterations in macrophage glycolysis, suggesting a link between the proinflammatory state and increased glycolysis levels. In investigating the underlying mechanisms, Zhang et al. (2021) concluded that BPF may affect macrophage glycolysis and consequently influence cytokine secretion via the PI3K-AKT pathway. It was also shown that the ER antagonist ICI 182,780 suppressed the secretion of proinflammatory cytokines in macrophages upon concomitant BPF exposure by inhibiting PI3K-AKT, indicating the involvement of ER (Zhang et al. 2021). Shi and colleagues (2020) also showed that estrogen-like BPF behaviour led to activation of the JAK2/STAT3 pathway, demonstrating the role of ER in M1 polarization of macrophages (Shi et al. 2020). The role of imbalanced macrophage polarization in disease development has been previously established; for example, an abnormal M1/M2 ratio is implicated in poor cancer prognosis, various autoimmune diseases, and obesity (Funes et al. 2018). Therefore, previous data suggest that BPF, through its estrogenic effect, may influence the shift toward proinflammatory macrophage polarization and potentially lead to the development or exacerbation of pathological conditions. The involvement of ER in the proinflammatory effects of BPF on macrophages was also demonstrated by Shi and colleagues (2020), who showed that BPF promotes the expression of SOC3 via the JAK2/STAT3 pathway, which is under the control of the ER receptor (Shi et al. 2020).

#### Dendritic cells

Dendritic cells (DCs) are best known for their antigen-presenting role, introducing foreign antigens into cells of the adaptive immune system and trigger primary immune responses. In addition, DCs are also an important factor in the development of immune tolerance to self-antigens, thus preventing autoimmune responses (Banchereau et al. 2000; Svajger and Rozman 2014). Several studies have already highlighted the potential effects of xenobiotics on aberrations in the complex life cycle of DCs, which may lead to impaired immune function.

While studying the effects of BPA and its analogues BPF and BPAF on DCs transformation from monocytes, Švajger et al. (2016) showed that bisphenols can affect the expression of differential cell markers during DCs maturation, where 30 µM BPAF significantly decreased the expression of CD1a, whereas the dendritic cell-specific intercellular adhesion molecule-3-grabbing non-integrin (DC-SIGN) was significantly upregulated by both BPA (50 µM) and BPAF (30 µM), whereas the CD14 marker remained more or less unaffected (Fig. 1). The evaluation of differential cell markers represents a suitable tool to determine the success of transformation into mature DCs (mDCs), which highly express CD1a and DC-SIGN, while the macrophage marker CD14 is present only to a very low extent. Based on the above results, the authors conclude that BPAF does not inhibit the maturation of DCs, but initiates the development of a specific population of mature DCs with low CD1a and high DC-SIGN incidence (Švajger et al. 2016). Interestingly, two other studies using the classical in vitro method of DC maturation obtained opposite results for the incidence of membrane markers after BPA exposure, namely increased CD1a expression (Guo et al. 2010; Camarca et al. 2016). This could be due to differences in the in vitro differentiation protocol—Švajger et al. (2016) exposed monocytes to bisphenols prior to exposure to differentiating stimulants, taking into account the direct effect of BPA on the process of monocyte differentiation (Švajger et al. 2016). It is also important to note the variability between the bisphenol concentrations used in the studies—Švajger et al. (2016) observed the above-mentioned bisphenol effects at 30 and 50 µM, while Camarca et al. (2016) and Guo et al. (2010) worked in much lower concentration ranges (0.1–1 nM and 0.01–0.1 µM, respectively) (Švajger et al. 2016; Guo et al. 2010; Camarca et al. 2016).

Švajger et al. (2016) also showed a significant effect of BPAF on DC maturation markers, with a twofold reduction in the expression of CD80 and CD86 markers, whereas the other xenoestrogens tested (BPA, BPF, and E2) showed no significant effect on the above markers (Fig. 1). The reduction of CD80 and CD86 markers might be related to decreased phosphorylation and thus activation of NF-ĸB and ERK1/2 signaling pathways in DCs activated with LPS after BPAF exposure (Švajger et al. 2016). It is already known that decreased NF-ĸB activity leads to decreased DC maturation, and furthermore, simultaneous downregulation of both NF-ĸB and ERK1/2 signaling pathways may further enhance the inhibition of DC maturation (Švajger et al. 2016; Peng et al. 2012). It is worth noting that increased numbers of CD86 molecules on the surface of antigen-presenting cells are associated with stimulation of allergen-responsive Th2 cells, which may be mediated by the aryl hydrocarbon receptor (AhR), which can be part of both an inflammatory and an immunosuppressive state. AhR agonists have previously been shown to induce CD86 and CD83 expression in DCs (Aguilera-Montilla et al. 2013). Moreover, Švajger and colleagues (2016) also observed significant changes in morphology in DCs pretreated with BPAF after activation with LPS (Fig. 1). For example, they noted an almost complete absence of distinctive cluster formation and dendrites structuring of mature DCs. In addition, BPAF was found to significantly impair the allostimulatory function of both mature and immature monocyte-derived DCs (Švajger et al. 2016). The recent findings raise concerns about the inherent safety of the BPA substitute BPAF, which may have potentiating immunosuppressive effects by impairing DC maturation and thus proper function DC.

#### Neutrophils

Neutrophils are an important player in the proper functioning of the immune system and perform several specialized functions. They are known for their role in the innate immune system and play a crucial role in acute inflammation (Kolaczkowska and Kubes 2013). Impaired function of any of the various neutrophil functions (such as chemotaxis, aggregation, adhesion, etc.) can significantly reduce host protection against pathogens and weaken the immune response (Bogomolski-Yahalom and Matzner 1995). Since neutrophils constitute the largest fraction of circulating human leukocytes, it is useful to study the effects of environmental pollutants such as bisphenols on their function.

Peillex and colleagues (2021) investigated how BPA, BPS, and their conjugated metabolites affect neutrophil metabolism and antimicrobial properties. The results showed a non-monotonic response to BPS and its conjugate BPS -glucuronate that decreased basal metabolism at concentrations of 1–10 pM and 1–100 µM and conversely increased glycolysis at a concentration of 20 µM (Fig. 1). However, a response was observed only with short-term exposure of BPS and BPS -glucuronate to purified human neutrophils. Interestingly, BPA and its glucurinated metabolite showed no significant effect (Peillex et al. 2021). Glycolysis is one of the main energy sources of neutrophils, so its impaired function may affect the proper function of neutrophils and thus possibly immune function (Jeon et al. 2020). The link between altered neutrophil metabolism and proper function was further demonstrated by exposing bisphenol-treated neutrophils to N-formylmethionine-leucyl-phenylalanine (fMLP), which resulted in an increase in glycolysis after long-term bisphenol exposure, followed by an overall decrease in neutrophil metabolism (Peillex et al. 2021). The study also observed a significant decrease in cytokines secreted by neutrophils, particularly CXCL8/ IL -8, after bisphenol exposure, with the results again following a nonmonotonic trend (Fig. 1). It is important to note that BPA and the glucurated metabolites of BPS more markedly decreased the concentrations of secreted cytokines (Peillex et al. 2021).

#### Monocytes

Monocytes account for approximately 10 % of circulating peripheral leukocytes in the human body and are known to mobilize rapidly to sites of inflammation, where they can differentiate into tissue macrophages or DCs or exert effector or regulatory activity independently as distinct monocyte subpopulations. As they exert proinflammatory properties and have been shown to play a key role in some autoimmune diseases, they are an important target of immunotoxicology research (Guilliams et al. 2018).

Buoso and colleagues (2021) studied THP-1, a human promyelocyte cell line commonly used for in vitro studies of monocyte/macrophage function. The results obtained in this manner may indicate potential immunomodulatory effects in vivo (Chanput et al. 2014; Buoso 2021). The study shed light on the possible mechanism behind the immunotoxic effects of bisphenols by measuring the effect of BPA, BPAF, and BPS on the expression of RACK1, which is known to play a key role in the activation of immune cells and whose expression depends on a complex hormonal balance. The results showed that BPA and BPAF at a concentration of 10 µM downregulated the transcriptional activity of RACK1, while BPS at the same concentration (10 µM) induced RACK1 expression (Fig. 1). The results suggest that bisphenols at higher concentrations could contribute to aberrant immune function, with BPS predisposing cells to a more immunostimulatory state that may lead to the development of various autoimmune or allergic diseases, while BPA and BPAF could act in an immunosuppressive (Buoso et al. 2021). Nevertheless, studies focusing on the immunotoxic effects of bisphenol analogues on monocytes are lacking, which represents an opportunity for future studies.

### Adaptive immune system

The second major branch of the functional immune system is adaptive immunity, also known as acquired or specific immunity. In contrast to innate immune responses, adaptive immunity takes longer to respond to foreign challenges and has a significant targeting function and memory component responsible for durable protection (Moser and Leo 2010; Toskala 2014). The main protagonists of the adaptive immune response are white blood cells, lymphocytes, which are divided into T lymphocytes (which can exert cytotoxic, effector, and regulatory activity) and B lymphocytes (which are responsible for antibody production) depending on their function. Endocrine disruptors are known to have effects on the above two subsets of adaptive immunity (Nowak et al. 2019), and in the following sections we have compiled information on the effects of bisphenol analogues on these critical components of the immune system.

#### T lymphocytes

T lymphocytes are an important component of the adaptive part of the immune system, and consequently, dysfunction at any level of differentiation into subsets [e.g., CTL, Th1, Th2, Th9, fTh, Th17, and regulatory T cells (Treg) (Moser and Leo 2010; Chraa et al. 2019)] by endocrine disruptors such as bisphenols have significant consequences for normal immune function (Malaisé et al. 2020a; Malaisé et al. 2021; Dong et al. 2020; Gao et al. 2020; Sabuz Vidal et al. 2021).

Previous research with BPA has already demonstrated the immunotoxic potential of the endocrine disruptor, as BPA has been shown to impair the ratio between Th1 and Th2 subsets of T cells (Fig. 1) and also affect appropriate Treg differentiation and function (Gao et al. 2020). Impaired Th1/Th2 ratio may lead to alterations in cytokine release and thus affecting appropriate defense against pathogens or even contribute to the development of allergic conditions (Moser and Leo 2010). Gao et al. (2020) also showed that BPA can exert its inflammatory effects via induction of the PI3K/Akt/mTOR signaling pathway (Gao et al. 2020). In addition to BPA, Malaise et al. (2020) tested the effect of BPF and BPS -analogs on spleen T lymphocytes of mice, and in vitro assays resulted in a significant increase in IL -17 levels after exposure to environmentally relevant concentrations of 0.05 nM of BPA and BPF (Fig. 1). BPS, on the other hand, had no effect on the release of IL -17 from mouse splenic T-cells. Similar results were also obtained after treatment of siLP-derived T cells with 0.05 nM of BPA, BPF, and BPS, which significantly increased the levels of IL -17 as well as IL -22 at this low concentration (Fig. 1). Nevertheless, no significant changes in Th17 frequencies were observed after treatment of mouse spleen or intestinal T cells with BPA and BPF (Malaisé et al. 2020a). However, in further studies in mouse models, an increase in Th1 and Th17 abundance was observed in female mouse offspring after perinatal dermal exposure to BPA and BPF (both at a concentration of 50 μg/kg bw/day) prior to intestinal immune cell restimulation with anti-CD3 (Fig. 1). However, the results were not consistent among female and male mice offspring, suggesting a greater sensitivity of female mice offspring to BPA and BPF exposure during the perinatal period (Malaisé et al. 2021). In contrast to the in vitro mouse T-cell assays mentioned above, only the highest bisphenol concentration tested had an effect on human T cells, where exposure to 50,000 nM BPA, BPF, and BPS significantly decreased secretion of IL -17 and IL -22 (Fig. 1). The bisphenols tested also caused a slight decrease in the percentage of IL -17 and IL -22 positive human T lymphocytes (Malaisé et al. 2020a). Secretion of IL -17 and IL -22 by Th17 lymphocytes appears to play a role in localized immune defense in the gut and lung and is also associated with autoimmune responses (Moser and Leo 2010). Disruption of various T lymphocyte subsets such as Th1, Th2, and Th17 by BPA and the analogs BPF and BPS can therefore potentially lead to impaired immune defenses against foreign organisms and contribute to the development of autoimmune diseases such as asthma.

#### B lymphocytes

Lymphocytes B are a part of the adaptive immune system and serve as the center of the humoral response, with the main functions of antibody production, secretion of inflammatory cytokines, antigen presentation, and intercommunication with lymphocytes T (Affara et al. 2014).

In a study by Jang et al (2020), the toxic effects of BPA and its substitutes on human WiL2- NS lymphoblast B cells, which have been shown to express the ESR1 receptor, were tested in vitro. The tested compounds decreased cell viability at a concentration of 100 µM, i.e., BPA decreased cell viability by approximately 70%, BPS by 20%, and BPF by 30% (Fig. 1). Further measurements by flow cytometry revealed that 26% of cells were in the apoptotic sub-G0/G1 region in the BPA- treated group, which was significantly higher than the 10% and 12% in the BPS and BPF groups, respectively, suggesting that BPA is more toxic to human B cells than its analogues (Jang et al. 2020). For comparison, a major in vivo study CLARITY-BPA examined the effects of BPA on components of the immune system in Sprague-Dawley rats and found that a daily dose of 25 μg BPA/kg bw resulted in a reduction in splenic lymphocyte B in female rats after six months of exposure (Li et al. 2018a). Moreover, they observed an increase in IgM-high cells among splenic lymphocytes B in female rats at postnatal day 90 (PND90) after daily BPA treatment with 25000 μg BPA/kg bw (Fig. 1). However, the authors emphasise that the results are sporadic in nature and occurred only in one group of the tested dosage (Li et al. 2018a; Li et al. 2018b). Further research is needed to adequately extrapolate these effects with potential implications for immune system function.

#### Peripheral blood mononuclear cells (PBMC)

Peripheral blood mononuclear cells (PBMCs) are a representative model for studying immune processes in the peripheral blood circulation, where contact between foreign chemicals, including endocrine disruptors, and cells of the immune system occurs most frequently. This is precisely the reason for the frequent use of PBMCs as a model in toxicological studies, where the response reflects the behavior of a combination of immune cells such as lymphocytes, monocytes, and macrophages (Pourahmad and Salimi 2015).

Research using PBMCs as cell models is based on the isolation of leukocytes, the so-called buffy coat, from human whole blood after treatment with separating agent (e.g., Ficoll) and centrifugation (Pourahmad and Salimi 2015; Michałowicz et al. 2015). Investigating the effects of different bisphenol analogues on PBMC integrity, Michałowicz et al. (2015) found that almost all bisphenols tested (BPA, BPF, and BPAF) had a negative impact on cell viability, with statistically significant differences observed at high concentrations (150 and 300 µM in the case of BPAF and 220 and 440 µM in the case of BPA) (Fig. 1). BPF showed cytotoxic potential only after a longer incubation period (4 hours), even at a high concentration of 500 µM. BPS had the least pronounced effect and showed only a slight decrease in cell viability at higher concentrations (2 mM). In the case of prolonged exposure (4 hours) of PBMCs to BPAF, statistically significant effects on viability were observed at concentrations as low as 3 µM. The authors pointed out the possible relationship between reduced cell viability and ATP deficiency after bisphenol exposure, which coincided with the greatest reduction in cellular ATP supply by BPAF. BPAF reduced ATP content in PBMCs at 1.5 µM (1h incubation) and 0.3 µM (4h incubation) (Fig. 1). A statistically significant decrease in ATP level was also observed in BPA and BPF (Fig. 1) at concentrations of 22–440 µM for BPA and 25–500 µM for BPF after 1h incubation and 2.2 µM (BPA) and 2.5 µM (BPF) after 4h incubation. Again, BPS had the least effect and decreased ATP levels at 80–400 µM (1h incubation) and 20–400 µg/ml (4h incubation) (Michałowicz et al. 2015). Similarly, Mokra and Michałowicz (2015) investigated the effects of the above bisphenols (BPA, BPF, BPS, and BPAF) on the occurrence of necrotic and apoptotic PBMCs and found that BPAF caused the greatest increase in necrotic cells by 75.2% at 100 µg/mL compared with the control, whereas a statistically significant increase was observed at 0.5 µg/mL. BPS showed the least effect in increasing the number of necrotic cells, whereas, on the contrary, it had the greatest effect on the increase of apoptotic PBMCs at a concentration of 20 µg/mL. The authors showed that the tested bisphenols affect the induction of programmed cell death via increased cytosolic Ca^2+^ levels and a decrease in transmembrane mitochondrial potential (Fig. 1). They are also involved in the activation of caspases (especially caspase 8, 9, and 3), which are important players in programmed cell death, and also affect PARP-1 cleavage (Mokra and Michałowicz 2015). It should be noted that bisphenols have been shown to alter the viability of PBMCs at very high concentrations to which ordinary people are not usually exposed in their daily lives. Nevertheless, it is worth noting that the concentrations of bisphenols that affect viability decrease significantly with long-term exposure. Therefore, the potential toxicity for PBMCs at daily exposure and the possible saturation of detoxification pathways should be considered.

Xenobiotic-induced cell death is usually associated with changes in cell morphology, and BPAF at a concentration of 3 µM and an incubation time of 4 hours also showed the greatest effects, resulting in decreased size of PBMCs and increased granulation (Fig. 1). The study also showed an increase in the formation of ROS in PBMCs after exposure to the bisphenols tested, ranging from 0.3 to 250 µM, with BPF exerting the strongest effect (2.5–25 µM) (Fig. 1). The results were also consistent with BPF having the greatest effect on lipid peroxidation at concentrations of 0.5 µM (1h incubation) and 0.1 µM (4h incubation) (Fig. 1), followed by BPAF (0.3 µM—1h incubation, 0.6 µM—4h incubation) and BPA (0.44 µM—1h incubation, 0.88 µM—4h incubation). Moreover, it was shown that BPA and BPAF caused significant protein damage after a 4-h incubation period (Fig. 1) (Michałowicz et al. 2015).

In comparison with the study conducted by Michałowicz et al. (2015), Mokra et al. (2018) determined a similar degree of immunotoxicity of bisphenol analogues by observing DNA damage after exposure to BPA, BPS, BPF, and BPAF. Consistent with the findings of Michałowicz et al. (2015), BPAF also exhibited the highest genotoxic potential (Fig. 1), while BPS again had the lowest effect on DNA damage (Michałowicz et al. 2015; Mokra et al. 2018). The authors suggested that the greater oxidative damage to DNA in the case of BPAF was mainly due to its electronegativity and reactivity due to additional trifluoromethyl groups, while the lower reactivity of BPS was due to its sulfonyl group and associated higher polarity (Michałowicz et al. 2015; Molina-Molina et al. 2013). BPA and BPAF caused concentration-dependent oxidation of both purines and pyrimidines at concentrations ranging from 0.01 to 0.1 µg/ml, whereas BPF and BPS significantly induced oxidation of only purine bases at concentrations of 0.01 and 0.1 µg/ml. The genotoxic properties of bisphenols are attributed to the formation of hydroxyl radicals (HO–) and their reactivity with DNA bases (Michałowicz et al. 2015). The authors emphasised that the tested bisphenol analogues BPA, BPAF, and BPF appeared to have toxic effects on PBMC DNA even at very low concentrations of 1 ng/mL, and most of the DNA damage was caused by single-strand DNA breaks (Mokra et al. 2018).

Overall, bisphenols affect the viability of PBMCs by impairing cellular ATP production, increased Ca^2+^ levels, and altered mitochondrial membrane permeability. BPA and its analogues also lead to changes in PBMC morphology and ROS production, leading to oxidation of cellular components (e.g., lipids and proteins) and causing genotoxic effects.

## Other immunomodulation-related effects of BPA analogues

### Effects on immune function-related gene expression

Previous studies have already shown mainly indirect effects of endocrine disruptors on gene expression through various epigenetic mechanisms such as DNA methylation, histone (de)acetylation, and RNA interfering (Xu et al. 2016). Micro RNAs (miRNAs) are known post-transcriptional gene silencers with the ability to bind to multiple complementary mRNA molecules, thereby down-regulating further translational processes (Gomes et al. 2013; Hausser and Zavolan 2014). Several endocrine disrupting chemicals, including BPA, can disrupt epigenetic processes, potentially influencing the development of pathological conditions (Hou et al. 2012; Bollati and Baccarelli 2010). It was found that BPS at concentrations of 5 µg/L and 50 µg/L can modulate the expression of 14 miRNAs in male zebrafish gonads. The aforementioned miRNAs have been shown to be involved in the development of the immune system and lymphoid organs as well as hematopoiesis (Lee et al. 2018). Current research also highlights the negative relationship between specific miRNA and expression of target genes (Lee et al. 2018; Wang et al. 2013). For instance, overexpression of the cyp19 gene, which encodes the aromatase enzyme responsible for converting androgen to estrogen (Sowers et al. 2006), was found in zebrafish exposed to BPS (Ji et al. 2013). It has also been shown that overexpression of cyp19a1 is associated with lower levels of dre-miR-30c, -430a, -430b, -192, 454b, and -499 miRNAs (Lee et al. 2018).

Another in vivo study by Dong et al. (2018) assessed effects of parental exposure to BPA (0.1 µg/L, 1 µg/L, and 10 µg/L), BPS (10 µg/L), and BPF (10 µg/L) on zebrafish F1 offspring with/without continued bisphenol exposure, focusing on the expression of Toll-like receptors (TLR) and their downstream signalling molecules, which eventually lead to the expression of cytokine- and chemokine-related genes. After treatment of control larvae with PAM3CSK4 and Poly I:C, there was a corresponding increase in the expression of TLR2 and TLR3 genes. The opposite was observed in the F1 larvae generation, where parental exposure to bisphenols resulted in decreased TLR gene expression, indicating a possible effect of bisphenols on impaired pathogen recognition. The study also found that parental exposure to bisphenols affected decreased expression of downstream immune signaling molecules such as MyD88 and TRIF-dependent signaling pathways, leading to decreased immunocompetence and consequently decreased survival of F1 larvae (Dong et al. 2018).

Similarly, Qiu et al. (2018a) investigated the effect of BPA and its analogues BPS and BPF on the expression of immune-related genes in zebrafish embryos, as shown in Table 2. The results show a concentration-dependent increase in the expression of immune genes, with the highest concentrations of BPS and BPF (1000 µg/L) increasing the mRNA levels of all cytokines/chemokines tested (Table 2). BPF had the strongest effect on increased expression; in comparison, BPA at a concentration of 100 µg/L was also shown to induce expression of almost all genes tested. In addition, suppressed effects of the above immune function-related genes were observed in the presence of estrogen receptor and NF-ĸB antagonists, shedding light on the potential mechanism behind gene expression regulation (Qiu et al. 2018b).

**Table 2:** Effects of BPS and BPF on the expression of immune-relevant genes in the concentration range of 0.1–1000 µg/L, compared to 100 µg/L BPA

mRNA	BPA 100 µg/L	BPS 0.1 µg/L	BPS 1 µg/L	BPS 10 µg/L	BPS 100 µg/L	BPS 1000 µg/L	BPF 0.1 µg/L	BPF 1 µg/L	BPF 10 µg/L	BPF 100 µg/L	BPF 1000 µg/L
*il-1β*	↑	–	–	–	↑	↑	–	–	–	↑	↑
*il-6*	↑	–	↑	↑	↑	↑	–	↑	↑	↑	↑
*il-10*	↑	–	–	–	–	↑	–	–	–	–	↑
*il-11α*	↑	–	–	–	–	↑	–	–	↑	↑	↑
*il-12α*	↑	–	↑	↑	↑	↑	–	–	–	↑	↑
*ifn γ*	↑	–	–	↑	–	↑	–	–	↑	↑	↑
*tnf α*	↑	–	–	–	↑	↑	–	–	–	–	↑
*cc-chemokine*	–	–	–	–	–	↑	–	–	–	↑	↑

Up arrows demonstrate induction of expression, while a dash indicates no significant change compared to control. Summarised after [Qiu et al. (2018a, b)]

The above studies highlight the vulnerability of aquatic organisms to environmental pollutants and indicate similarities in the in vivo immunotoxic effects of bisphenol analogues such as BPF and BPS with those of BPA, which may contribute to discrepancies in zebrafish survival and development.

In addition, we have examined in vitro studies focusing on altered expression of immune-related genes upon exposure to bisphenol analogues. For example, in the study by Fic and colleagues (2015), the effect of BPA, BPAF, and BPS on the expression of immune-related genes was observed, particularly the downregulation of chemokine-related genes, with the strongest effect observed when the human osteosarcoma cell line (HOS) was treated with BPS (10 nM) after three months of exposure. The most remarkable reduction in chemokine-related gene expression was observed for chemokine ligand 2 (CCL2), a known chemoattractant in numerous immune cells, such as macrophages, monocytes, B and T cells, basophils, DCs, and NK cells. It is involved in many immune diseases such as allergic asthma, psoriasis, and rheumatoid arthritis, and its downregulation was observed in all three bisphenols tested, with BPS having the greatest impact (Rose et al. 2003; Inadera et al. 2000; Fic et al. 2015). It is worth noting that the exposed HOS cells in this specific study were categorised as oestrogen-dependent cell lines and treated with the above-mentioned bisphenols at a concentration of 10 nM, which falls within the range of BPA concentrations detected in human biological samples, thus highlighting the true immunomodulatory potential of BPA and its analogues (Fic et al. 2015; Vandenberg et al. 2007).

### Oxidative stress

Oxidative stress is the result of an imbalance between antioxidant and pro-oxidant processes that occur as a by-product of cellular activities, and also plays an important role in the normal functioning of the immune system. For example, the production of ROS during phagocytosis in fish immune defenses plays a critical role in the subsequent formation of free radicals and potent bactericides that contribute to the elimination of pathogens (Biller and Takahashi 2018). In view of the increasing bisphenol concentrations in the aquatic environment, some published studies indicate the harmful effects of BPA on the immune system of fish through oxidative stress (Xu et al. 2013; Qiu et al. 2016; Wu et al. 2011). In addition, Qiu et al. (2018a) showed that the BPA analogues BPS and BPF at a concentration of 1000 µg/L caused a significant induction of the formation of ROS in zebrafish embryos, as well as a concentration-dependent increase in other oxidative stress markers such as SOD (superoxide dismutase) and LPO (lipid peroxidation), and T-AOC activity (total antioxidant capacity). Moreover, the increase in NO levels was also observed after exposure of zebrafish embryos to 100 and 1000 µg/L BPS and BPF. Overall, Qiu et al. (2018a) showed that BPS and BPF affect the immune system of zebrafish in a similar manner as BPA, raising questions about their actual safety in the aquatic environment (Qiu et al. 2018b). Interestingly, another study by Dong et al. (2018) that examined the expression of oxidative defense-related genes in zebrafish offspring after parental exposure to BPA, BPS, and BPF came to contradictory results. They found that parental exposure to bisphenols significantly downregulated the expression of key oxidative protection genes, such as MPX, CAT, Cu/Zn- SOD, and Mn- SOD. It is worth noting that Dong et al. (2018) used much lower bisphenol concentrations, with the highest concentration being 10 µg/L (Dong et al. 2018). In this regard, the controversy between the studies can be attributed to a possible biphasic effect of bisphenols, as already mentioned in the literature in the case of BPA (Ge et al. 2014). These results therefore suggest that parental exposure to low levels of bisphenols leads to less protection against ROS in the F1 generation, whereas high levels of bisphenols result in a concentration-dependent increase in the formation of ROS and related markers in zebrafish embryos.

The effect of bisphenols on the increase in the formation of ROS was also found in human PBMCs, with BPAF standing out significantly and causing the highest increase in the formation of ROS, followed by BPF. A study by Michałowicz et al. (2015) showed that BPA and BPS have the greatest effects on protein damage and consequently on their proper function via oxidative stress, while BPAF plays the greatest role in the formation of lipid peroxidation. The authors of the study showed that bisphenol analogues can cause oxidative stress in PBMCs at concentrations as low as 0.06–0.5 µM, which are already concentrations that can occur in humans due to occupational or environmental exposure (Michałowicz et al. 2015). Furthermore, Jang et al. (2020) demonstrated a significant 9.5-fold increase in superoxide dismutase 2 (SOD2) gene expression in the human B lymphoblast cell line WiL2- NS after a 4-h incubation with 100 µM BPA, and subsequent analysis revealed a similar significant increase in SOD2 gene expression after cell treatment with BPS and BPF (Jang et al. 2020). Although the SOD2 gene is not one of the primary representatives of genes related to the immune system, it is an important player in the regulation of the innate immune system, as its dysfunction leads to, among other things, increased replication of viral RNA (Wang et al. 2017).

### Intestinal immune system

BPA is one of the most well-known contaminants in the food industry that affects lymphoid tissue in the intestine through food. Lymphoid tissue in the gut is tightly intertwined with cells of the innate and adaptive immune systems as well as the gut microbiota (Xu et al. 2016a; Malaisé et al. 2020c). Studies on BPA suggest altered tolerance to food antigens, gut dysbiosis, and immune system imbalance that may lead to metabolic disorders and altered glucose metabolism (Malaisé et al. 2017; Menard et al. 2014; Lai et al. 2016; Ménard et al. 2014).

The human microbiome, while important, is also an underappreciated factor in the proper functioning of the immune system. Microbial metabolites are thought to play a role in regulating the immune system, and imbalances in this area can lead to the development of various diseases (Levy et al. 2017). Studies show that the aforementioned microbial metabolites communicate with mucosa-associated invariant T cells (known as MAIT), which may be susceptible to the effects of various food contaminants such as bisphenols (Corbett et al. 2014). Acute exposure of *Bacteroides thetaiotaomicron* and *Escherichia coli*, a part of the human microbiome, to doses relevant to tolerable daily intake (2.3 μg/mL, 28.3 μg/mL, and 354.0 μg/mL) of BPA, BPF, and BPS, showed effects on microbial growth, with the greatest effect on Escherichia coli with BPF exposure, whereas the growth of *Bacteroides thetaiotaomicron* was most affected with BPA exposure (Krause et al. 2022). Similarly, prenatal exposure to 50 μg/kg body weight (bw) BPS was shown to result in significant induction of anti-*E. coli* IgG in mouse offspring (Malaisé et al. 2020c), which was consistent with other studies in which pregnant female mice were exposed to BPA, BPS, and BPF at concentrations of 50 μg/kg bw/d via the skin (Malaisé et al. 2021). BPA had the greatest effect on microbiome metabolism at doses as low as 28.3 μg/ml. In terms of affecting the human microbiome, its growth, metabolism, and viability, the metabolites of BPF and BPS had lesser effects, possibly making them somewhat safer options. A study by Krause et al. (2022) also found reduced MAIT cell activation prior to bisphenol exposure, with BPA having the greatest effect, followed by BPF and BPS (Krause et al. 2022). According to the above research, exposure to bisphenols has the potential to alter microbial metabolism and directly affect immune cells.

In an in vivo study conducted by Malaise et al. (2020), adverse effects on gut immune responses were observed after prenatal oral administration of BPA and its analogs BPS and BPF at doses of 5 and 50 μg/kg body weight (bw) to pregnant female mice. BPA and BPF at a dosage of 5 μg/kg body weight affected the intestinal and systemic immune response of mouse offspring by disrupting the Th1/Th17 balance, while BPS at any dosage increased fecal lipocalin levels (Malaisé et al. 2020c). Lipocalin has already been established as a sensitive biomarker of intestinal inflammation, and the effect of BPS on lipocalin levels was replicated in the subsequent study, which found an increase in lipocalin levels along with a decrease in fecal IgA following perinatal dermal exposure to BPS in mice (Malaisé et al. 2021; Chassaing et al. 2012). The association between induced lipocalin levels in feces and the decrease in IgA concentration has been previously highlighted and linked to intestinal inflammation (Malaisé et al. 2021; Toyonaga et al. 2016). In addition, it was found that even low levels of all bisphenols tested (BPA, BPS, and BPF) resulted in a decrease in fecal IgA levels in female mouse offspring (Malaisé et al. 2021). On the contrary, adult male offspring exhibited increase in fecal IgA levels, suggesting sex-specific effects. As intestinal IgA plays a crucial role in regulating the balance between the host immune system and commensal microbiota, these results are significant (Malaisé et al. 2021).

Accordingly, Brulport and colleagues (2021) also demonstrated the effects of BPS on intestinal inflammation in mice after perinatal exposure. They found that parental and current exposure to BPS affected the release of TNF-α and lipocalin in male F1 offspring, which continued into the F2 generation. Finally, the levels of inflammatory markers decreased in the F3 generation, except for IFN-γ, which was higher in the BPS group. Because additional metabolic changes were observed in the unexposed F3 group, the authors raised concerns about possible transgenerational transmission of BPS induced epigenetic changes. The authors also observed sex differences, such as a decrease in gut inflammatory response in female F1 offspring and, conversely, an increase in inflammation in the F3 generation (Brulport et al. 2021).

In addition, Malaisé et al. (2021) found that cytokine release profiles differed by sex after perinatal skin exposure to BPA, BPS, and BPF. They demonstrated increased levels of IFN-γ in the supernatant of siLP (small intestinal lamina propria) in female mice offspring after perinatal exposure to BPS (5 μg/kg bw/day) and BPF (50 μg/kg bw/day). Conversely, these concentrations caused a decrease in IFN-γ release in male mice offspring. The IL -17 release rate followed a similar trend after perinatal exposure to 50 μg/kg bw/day BPF—showing increased cytokine release in female offspring and reduced IL -17 secretion in male offspring compared with control (Malaisé et al. 2021).

Considering the studies mentioned in this chapter, bisphenol exposure may alter microbial metabolism and thus affect immune cells. They are also capable of immunomodulating intestinal immune processes in mouse offspring after perinatal exposure, representing a possible transgenerational transmission of epigenetic changes in mice. Studies also show sex differences in measured effects, suggesting sex-specific responses.

### Outcomes related to immune system, such as asthma and allergy-related diseases

Reports of occupational occurrence of allergic contact dermatitis in industry are accumulating in workers exposed to epoxy resin monomers, which constitute the majority (75–90%) of polymers of diglycidyl ethers based on bisphenol A (DGEBA/BADGE) and in some cases bisphenol F (DGEBF). Both of the above compounds have a high sensitizing potential and are therefore classified as highly allergenic (O’Boyle et al. 2012).

Accordingly, several human birth cohort studies have reported an association between prenatal BPA exposure and allergy symptoms such as asthma and wheezing in children at different ages (Gascon et al. 2015; Spanier et al. 2012; Donohue et al. 2013). Yanagisawa and colleagues (2019) have shown that BPA can influence the increase in allergic inflammatory symptoms by stimulating Th1/Th2 chemokine expression and increasing OVA -specific immunoglobulin production. They also showed that OVA + BPA exposure affects lung hormone receptors by reducing Erb and Ar mRNA levels, while having no effect on Ahr, GR, and ESRRG (estrogen-related receptor gamma) (Yanagisawa et al. 2019). Similarly, a paper by Guo et al. (2010) found a link between Th2 deviation caused by stimulation of specialized DCs by BPA in the presence of TNF-α and allergic reactions (Guo et al. 2010).

Although many articles mention allergen effects as another way to demonstrate the immunotoxicity of BPA, there are currently not many studies investigating the sensitization effects of BPA analogues. Therefore, Mendy et al. (2020) analysed National Health and Nutrition Examination Survey (NHANES) data from 2013 to 2016 and concluded that neither BPS nor BPF is a safe BPA substitute. The authors found a positive correlation between urinary BPF levels and current asthma and hay fever, while BPS was associated with a higher likelihood of asthma in men. Nevertheless, further prospective studies are needed to determine the allergenic properties of other bisphenols besides BPA (Mendy et al. 2020).

### Autoimmunity

Previous research has already established a complex relationship between estrogen and the immune system (Steinmetz et al. 1998). BPA, a known xenoestrogen, has been linked to development of autoimmune diseases through multiple mechanisms. For example, increased urine BPA levels are positively correlated with hyperprolactinemia, which is linked to various autoimmune diseases such as multiple sclerosis, SLE, antiphospholipid syndrome, rheumatoid arthritis, thyroid and celiac disease, and diabetes mellitus type I (Aljadeff et al. 2018). Again, based on the structural similarity and evidence of comparable effects to BPA in other aspects of the immune system mentioned above, it is also suspected here that exposure to BPA analogues may predispose to autoimmunity, especially during the gestational period when the immune system is under development (Aljadeff et al. 2018).

However, a study by Xu et al. (2019a) found contradictory results in the case of BPS, when they examined its effects on the development of type I diabetes in nonobese diabetic (NOD) mice. In contrast to BPA, they found no significant effects on immune processes after BPS exposure in mice fed a soy-based diet. In the study, adult female mice were administered environmentally relevant BPS concentrations of 3, 30, 150, and 300 µg/kg, while male mice were treated with 0 or 300 µg/kg BPS. Although minimal effects were observed in terms of immunotoxicity, BPS was found to alter blood glucose levels in mice on soy-based diet, likely due to mechanisms beyond immunomodulation. Different dietary groups in male mice (soy-based and non-soy-based) clarified the possible interactions of BPS and phytoestrogens, as male mice on non-soy-based diets showed protective effects on blood glucose levels, while males on soy-based diet initially exhibited some degree of adverse effects, such as altered glucose homeostasis and heightened insulin resistance (Xu et al. 2019a). The results of the above study contrast with similar studies that examined BPA and found progression of type I diabetes and/or proinflammatory effects in female NOD mice (Bodin et al. 2013, 2015; Xu et al. 2019b), suggesting different mechanisms of action in the case of BPS.

## Conclusion

Since the introduction of new regulatory dossiers based on the use of BPA in industry, an increasing occurrence of bisphenol analogues in everyday products and consequently in the environment has been observed (Ye et al. 2015; Chen et al. 2016). The bisphenol analogues are characterised by the presence of highly reactive hydroxyphenol groups linked by a bridge to which various chemical groups can be attached, giving the analogues a high degree of versatility. Given the content of similar structural elements, there is concern for similar, if not greater, potential toxic properties of BPA analogues, which have not yet been so well investigated (Usman and Ahmad 2019). In addition to the already demonstrated similar activity of some bisphenol analogues on nuclear receptors, such as ER, AR, GR, AhR, and PXR (Program and Protocol for Systematic Review of Bisphenol a (Bpa ) 2015; Program and Biological activity of Bisphenol A (BPA) structural analogues and functional alternatives 2017; Sui et al. 2012; Sui 2014; Ziv-Gal et al. 2013; Rosenmai et al. 2014; Kolšek et al. 2014), there is also a risk of potential immunotoxic effects, among others (Xu et al. 2016a; McDonough et al. 2021b). Studies to date have shown that BPA analogues can affect many components of the immune system, ranging from the building blocks of innate to adaptive immunity, and through this influence the aberrant functioning of the immune system, which manifests itself in different ways, such as occurrence of various hypersensitivity reactions and allergies, immunosuppression or even an exaggerated immune response, all depending on the concentration and route of exposure. In vivo and in vitro studies have also shown the impact of bisphenol analogues on the microbiome, which can contribute to inflammation in the gastrointestinal tract (Krause et al. 2022; Corbett et al. 2014; Brulport et al. 2021; Malaisé et al. 2020b). This can be particularly problematic as most people are exposed to BPA and its analogues via the oral route (Xu et al. 2016a; Malaisé et al. 2020c). Our literature review revealed that there is still much room to investigate the mechanisms behind bisphenol-mediated immunomodulation and that studies in the area of the adaptive immune system, particularly in B-cell models, are lacking. We also noticed absence in relevant studies on the effects of bisphenols on eosinophil function, which would also make an important contribution to the understanding of various immunological manifestations such as allergy. So far, the studies have shown the impact of BPA analogues on the expression of immune-related genes and epigenetic mechanisms, the production of reactive oxygen species (ROS), and interference with certain cell receptors and cell signalling pathways in immune cells (Salem 2004; Qiu et al. 2018c; Zhang et al. 2021; Švajger et al. 2016; Buoso et al. 2021; Jang et al. 2020; Xu et al. 2016; Lee et al. 2018; Dong et al. 2018; Rogers and Mirza 2013). However, given the characteristic biphasic action of bisphenols, interpreting the data and drawing meaningful conclusions can sometimes be challenging. It is as well crucial to emphasise that the data in this review include only a few of the most common BPA alternatives, but there are also a number of other, newer BPA analogues that are less studied and increasingly found in industry and consequently in the environment. Since humans come into contact with several bisphenol analogues and alternatives simultaneously, it would be worthwhile to study the effects of different mixtures and combinations on the components of the immune system to obtain a much more realistic picture of the immunotoxic activity of bisphenols as well as additional information on their interactions, which may be additive or even synergistic. We can conclude that it is still difficult to give an assessment of the immunomodulatory effects of BPA analogues due to data scarcity, because of the narrow range of concentrations tested, the limited number of analogues included in the studies, the sparse information on the mechanisms behind the sex-dependent effects, the lack of understanding of the interactions between bisphenols and other endocrine disruptors, and, most importantly, the lack of studies in the area of in vivo effects in humans and of epidemiological studies.

## Data Availability

All data produced or analyzed during this study are enclosed in this article. All visual material, such as included images, was created using BioRender.com licensing software.
